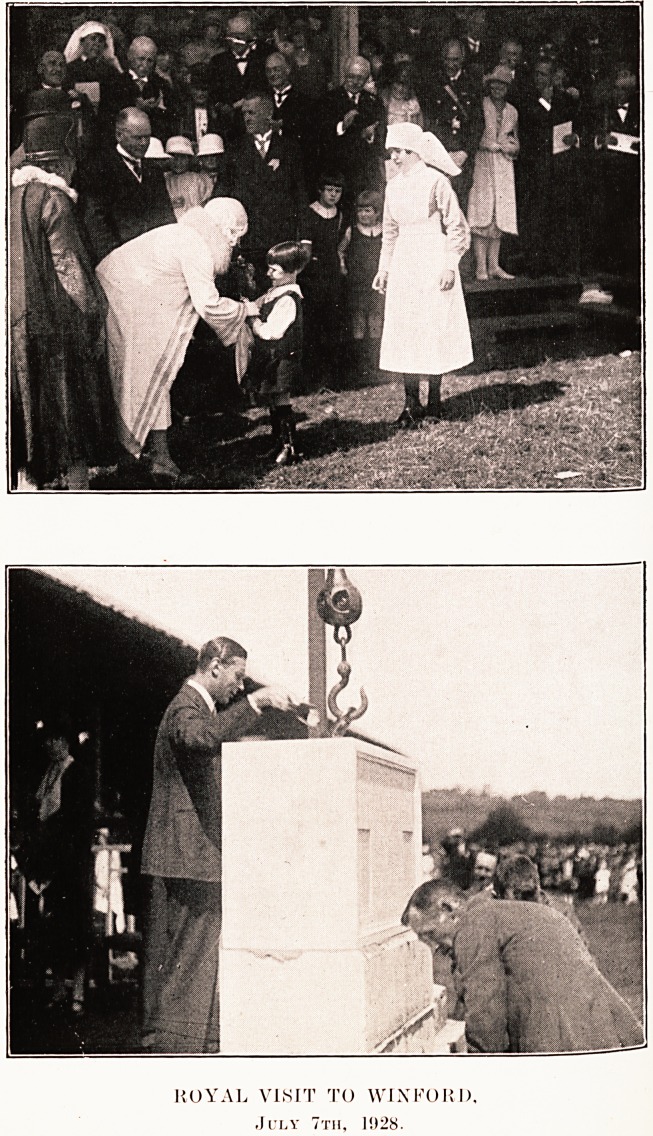# Editorial Note

**Published:** 1928

**Authors:** 


PLATE VI.

ROYAL VISIT TO WINFORD,

July 7th, 1928.

### Editorial Note.

**The Royal Visit to Winford.**

On July 7th the Duke and Duchess of York came to Bristol to lay the foundation stone of the new open-air hospital at Winford. The weather was beautifully fine, and—thanks to the excellent arrangements made by those in charge—a great many interested citizens were enabled to watch the ceremony and to see for themselves the beauty of the surroundings in which the new hospital is to be built. The purposes of this hospital were explained by Miss F. M. Townsend, J.P., Chairman of the Bristol Crippled Children's Society, in a little speech which was a model of compactness and lucidity. One point on which she laid particular emphasis deserves notice, and that is the example of union which this new movement sets to the other institutions of Bristol. The Bristol Crippled Children's Society and the Bristol Orthopædic Hospital have found it possible to sink their differences and pool their assets, and this ought to prove the first instalment of a larger and more general process of hospital union such as is taking place in most of the University towns of this country.

In this connection it should be noted that the site purchased is larger by far than the present needs of the open-air hospital demand. About seventy acres are available for the building of country annexes to the other voluntary hospitals of the city, if and when these are thought necessary. This site was chosen most carefully, geological and other experts being consulted, and it was in the belief that it was the best, from every point of view, that the neighbourhood of Bristol could furnish that it was chosen. (In passing it may be noted that the upper of the two pictures which we publish shows Sir Robert Jones applauding vigorously. This is by no means the only expression of his approval of the site. He visited it early in the negotiations for purchase, and was unequivocal in his praise.) Before the site was bought the principal officers of the chief voluntary hospitals of the city were consulted, and though they were not in a position to commit their institutions actively to the support of the movement, it was understood that they did not, in principle, disapprove of it. Before long it is probable that some one of these institutions will give a lead to the others by planning an extension of its accommodation in the form of a country annexe on the Winford site.

A third point of importance on which Miss Townsend dwelt was the fact that this country hospital embodies a form of union between voluntary and State effort which may prove to be a valuable precedent. The building of the hospital is in the hands of a voluntary committee, while the income which pays for its upkeep will be largely derived from Board of Education grants. It seems possible that this characteristically British plan of compromise may preserve for the hospital movement that freshness and originality which marked the voluntary era, while adding to this the stability that is ensured by State support.

Finally, Miss Townsend found it necessary to emphasise the fact that this movement needs a great deal of money. The present position is this, that the cost of the undertakings already entered upon is approximately £50,000. Towards this a sum of over £25,000 has been subscribed or promised. The appeal for subscriptions to meet this deficit will, we feel sure, be all the more successful because it is made on behalf of a movement that initiates a policy of hospital union in Bristol.

## Figures and Tables

**Figure f1:**